# Evaluation of orthodontic tooth brush designs among patients with fixed orthodontic appliances

**DOI:** 10.6026/973206300212356

**Published:** 2025-08-31

**Authors:** Marut Nandan Rai, Ashutosh Wadhawan, Yasir Ayub, Shipra Nagar, Aparna Rai, Ashish Kumar

**Affiliations:** 1 Department of Orthodontics and Dentofacial Orthopedics, Kalka Dental College, Meerut, Uttar Pradesh, India; 2 Department of Endodontics, Banaras Hindu University, Varanasi, Uttar Pradesh, India

**Keywords:** Gingivitis, plaque, bleeding, bonded bracket index, toothbrush

## Abstract

Many companies have developed toothbrushes customized to different age groups and patient needs. This research aimed to assess and compare the efficiency of four commercially available toothbrushes in eliminating plaque and controlling gingivitis in orthodontic patients. Total 40 subjects undergoing orthodontic treatment were assigned into four groups (n=10 per group): Group 1-Stim Manual, Group 2-Perfora Electric, Group 3-Oral B Powered, Group 4-Ultrasonic. Plaque Index, Gingival Index, Eastman’s Interdental Bleeding Index and Bonded Bracket Plaque Index assessed at baseline and at follow up visit were assessed. Thus, electric tooth brushes were the most effective in improving oral hygiene during fixed orthodontic treatment, followed by ultrasonic, powered and manual brushes.

## Background:

A beautiful smile is built on a foundation of healthy teeth and periodontal structure. It is essential for patients having fixed orthodontic procedure to practice good oral hygiene. Fixed orthodontic appliances are prone for plaque accumulation [1]. Hence maintenance of proper dental hygiene throughout orthodontic treatment is crucial to preventing cavities and periodontal disease [1]. Dental plaque is a biological layer that is arranged both structurally and functionally. It is the community of microorganisms that reside as a biofilm on the surface of teeth, surrounded by a host and bacterial polymer matrix [2]. Maintaining good oral hygiene is essential for anyone having fixed orthodontic procedure [3]. One of the most crucial things for people receiving orthodontic treatment is maintaining good oral hygiene [4]. Since orthodontic bands, ligature wires, brackets, and elastics promote the build-up of food particles and microbiological flora, which over time frequently exacerbates gingivitis, periodontal diseases, and white spot lesions on the coronal surfaces of teeth [5]. Even if there is debate over long- term data on the condition of orthodontic patients' periodontal tissues, this is still the case [6, 7-8]. Various designs of tooth brushes are developed to eliminate dental plaque. The manual orthodontic toothbrush Stim Ortho is one of many stylish orthodontic toothbrushes on the market. It comes with braces and special v-cut teeth-cleaning bristles [3]. There is a single tufted brush for cleaning under braces and along the gum line. The toothbrush on the other side of the handle is ideal for cleaning in between overlapping teeth and under braces [4]. Thermosealorthobrush is a manual, soft-bristled brush that was skillfully created. The long outside bristles, measuring 10 mm in length, gently massage gums and clean the tooth surface, eliminating plaque from the gum line, while the short interior bristles, measuring 9.5 mm in height, facilitate cleaning between braces and teeth. Each bristle is 8 mm in diameter and features a round end to protect gums and enamel [9].

An electric toothbrush has a rotating brush head [10]. This toothbrush provides deep cleaning and effectively eliminates plaque and promotes interdental cleaning with its 8800-rpm oscillating motion. Battery-Powered Oral-B Toothbrushes are designed to help you get a better clean by stimulating your gums and removing plaque [9]. Improved gum health through the elimination of plaque Bristles penetrate deeply to remove plaque between teeth and polish stains away for white teeth. An electronic toothbrush intended for regular usage at home is called an ultrasonic toothbrush that helps remove plaque and renders plaque bacteria harmless by creating ultrasonic waves [10]. It typically operates at 1.6 MHz, is equivalent to 96,000,000 pulses or 192,000,000 movements each minute. Numerous clinical and laboratory studies involving patients receiving fixed orthodontic treatment have examined the efficiency of various toothbrush types [9, 10]. Both manual and electric toothbrushes are equally good at cleaning, according to some research on non-orthodontic people, but other studies show that electronic toothbrushes are more effective. Therefore, it is of interest to assess and compare the efficiency of four commercially available toothbrushes in removing plaque and controlling gingivitis in orthodontic patients.

## Materials and Methodology:

The study was done at Kalka Dental College Meerut after obtaining the ethical clear from the institute and informed consent form the participants. This study included 40 subjects aged between 13 to 32 years of age visiting the Department of Orthodontics and Dentofacial Orthopedics for the fixed orthodontic treatment. Patients with at least 20 teeth in the oral cavity, at least 16 brackets or bands on teeth, brushing at least once a day, being between the ages of 13 and 32, not using antibiotics within the previous two months, and not having menstruation or pregnancy at the time of score recording were included. Patients who were undergoing fixed orthodontic treatment with upper and lower pre-adjusted edgewise appliance therapy concurrently were also included. The following are exclusion criteria: systemic disease; use of antibiotics, steroids, or nonsteroidal anti-inflammatory drugs (NSAIDs) within the last two months or during the study; fewer than five teeth per quadrant; immunosuppressive medication use; medically compromised patients; mentally challenged subjects; subjects with poor manual dexterity; subjects who received oral hygiene instructions from a dental professional within the last six months; severe gingival inflammation; absence of obvious systemic or local periodontal disease, attachment loss, or pocketing; use of antibacterial mouth rinses; juvenile/aggressive periodontitis; use of manual orthodontic toothbrushes in the past or present; and severely carious teeth pregnant women, smokers, and users of tobacco products.

40 patients of fixed orthodontic appliances were chosen. They were divided into 4 groups each consisting of 10 patients.

[1] Group 1- Stim ortho MB(N) = 10

[2] Group 2- Perfora electric toothbrush(N) = 10

[3] Group 3- Oral B (powered toothbrush) (N) = 10

[4] Group 4- Ultrasonic toothbrushes(N) = 10

The participants in the study were provided with fluoride-containing toothpaste without antiplaque or anti-calculus agents. They received oral hygiene instructions and demonstrations using plastic models of dental arches with orthodontic appliances. Using a timer, a tooth brushing booklet and a planner, they were told to brush for two minutes twice a day. Mouth rinses, dental floss, interproximal brushes, and other cleaning supplies were prohibited. Different types of toothbrushes were used; including a manual orthodontic brush, electric, powered and sonic toothbrushes with instructions based on manufacturer recommendations. Plaque assessment was done in all the patients, clinical examinations were done to assess gingival health, plaque, and interdental bleeding at baseline, 4 weeks, 8 weeks and 12 weeks. The assessment included the use of plaque and gingival indices, as well as interdental bleeding index. At follow-up visits, patients were also asked about any brushing-related trauma and plaque assessment was performed using a modified version of the Silness and Loe plaque index.

## Results and Discussion:

Version 17 of the Statistical Package for Social Sciences (SPSS) software was used to conduct the statistical analysis. Intragroup comparisons were conducted using the paired t-test. ANOVA, or one-way analysis of variance, was used to compare groups. A significance criterion of p < 0.05. In Table 1 average score of all indices is explained for groups using STIM orthotodontic tooth brush. Gingival Index decreased from 1.08 to 1.03, indicating reduced gum inflammation. Plaque Index declined from 1.14 to 1.04, reflecting improved plaque control. Table 1 (see PDF) indicates a significant decrease in the plaque index and GI scores between baseline and the 12-week follow-up. This might be because patients took some time to get used to using an orthodontic toothbrush following fixed appliance bonding, and the outer bristles are positioned at a 45° angle to the gum line. Additionally, this might be because of the head's size, which made it challenging to fit into spaces between teeth [11]. In Table 2 (see PDF) average scores of all indices is explained for groups using electric orthodontic tooth brush, it shows a progressive improvement in oral health over 12 weeks, as indicated by decreasing scores in all four indices. Gingival Index decreased from 1.08 to 1.01, showing reduced gum inflammation. Plaque Index dropped from 1.13 to 1.02, indicating better plaque control. EIBI fell from 0.18 to 0.05, reflecting significantly reduced interdental bleeding. BBPI improved from 1.14 to 1.03, suggesting enhanced brushing effectiveness. Average scores of all indices in groups using power orthodontic toothbrush as given in Table 3 (see PDF) shows a steady improvement in oral health over 12 weeks. Gingival Index reduced from 1.09 to 1.04, indicating less gum inflammation. Plaque Index decreased from 1.12 to 1.03, reflecting better plaque control. EIBI dropped significantly from 0.22 to 0.04, showing reduced interdental bleeding. BBPI declined from 1.09 to 1.01, suggesting improved brushing technique. Mean scores of all the indices for study groups using ultrasonic orthodontic toothbrush as given in table 6 shows gradual improvement in oral health from baseline to 12 weeks. Gingival Index decreased from 1.09 to 1.03, showing reduced gum inflammation. Plaque Index dropped from 1.12 to 1.02, indicating better plaque control. EIBI fell from 0.19 to 0.04, reflecting significantly less interdental bleeding. BBPI declined from 1.12 to 1.02, suggesting improved brushing effectiveness. On intergroup comparison, statistically considerable variation were found in GI, PI Eastman interdental bleeding index and BBPI index scores from baseline and 12 week for electric and ultrasonic toothbrushes when average variation from T0 to T1, T0 to T2, T1 to T2 to T2 to T3 were compared as seen in Table 4 (see PDF). But, highly statistical significant value was noted for electric toothbrush. This demonstrated that while electric and ultrasonic toothbrushes were both shown to be efficient in reducing gingivitis and plaque in patients receiving fixed orthodontic treatment, the electric toothbrush was the most successful. On the other hand, the visual plaque index (VPI) was statistically lower when using a manual orthodontic toothbrush. The gingival health of patients with fixed appliances improved in our study, even though four trials had lower plaque ratings (Table 5 - see PDF).

The efficiency of various toothbrush types has been compared in numerous clinical and laboratory investigations involving patients undergoing fixed orthodontic treatment. While some Studies on non-orthodontic patients show that electric toothbrushes are more effective at cleaning, other studies show that both manual and electric toothbrushes are similarly efficient. Only one study shows that manual and electric toothbrushes are similarly efficient, while most studies on orthodontic patients show that using electric toothbrushes improves periodontal health. These research' findings, however, were discovered to be contradictory [7]. In present study, on intergroup comparison, highly statistical significant value was noted for electric toothbrush. A related study by Sharma *et al.* (2015) [11] compared manual, powered, and sonic toothbrushes in 60 orthodontic patients and concluded that sonic brushes were superior in reducing gingivitis and plaque. This is consistent with our findings. Kalf-Scholte *et al.* (2018) [12] reviewed triple-headed versus single-headed manual brushes and found better plaque removal with triple-headed designs when used by caregivers. This supports our observation that brush design can influence plaque control outcomes. Grossman *et al.* [13] suggested that the sonic action of some electric brushes may enhance plaque disruption and reduce inflammation supporting our findings favouring electric brushes. Hickman *et al.* (2002) [14] found minimal differences in plaque control between powered and manual brushes. Similarly, Kilicoglu *et al.* (1997) [15] found no significant difference between orthodontic and regular manual brushes regarding plaque and gingivitis, although the comparison did not include electric or ultrasonic brushes. Thienpont *et al.* (2001) [16] conducted a crossover clinical trial with four toothbrush types (two electric, two manual) and found no significant differences in plaque or gingival scores. However, they noted that plaque removal was more capable in the lower jaw, highlighting potential anatomical factors in brushing effectiveness. Anas *et al.* (2018) [17] found that ultrasonic brushes performed better than manual ones in a study of 50 students. Borutta *et al.* (2002) [18] reported statistically significant improvements with powered brushes, although most such studies were short-term. In our study, no adverse effects such as tissue trauma or gingival abrasion were noted after 6 months, confirming the safety of all tested toothbrushes. On intragroup comparison, electric toothbrushes outperformed others in reducing plaque, gingivitis, bleeding, and bonded bracket plaque. Intergroup analysis ranked effectiveness as follows: electric > ultrasonic > powered > manual. Electric toothbrushes were found to be the most effective in improving oral hygiene in patients undergoing fixed orthodontic treatment, followed by ultrasonic, powered, and manual orthodontic brushes. Future research should include longer follow-up periods and explore newer toothbrush designs for optimal oral health outcomes in orthodontic patients.

## Conclusion:

The study compared 4 commercial tooth brushed types and concluded that, electric toothbrushes were the most effective in improving oral hygiene during fixed orthodontic treatment, followed by ultrasonic, powered and manual brushes. We recommend that, any of the 4 brushes can be recommended for orthodontic patients in order to maintain their oral hygiene during fixed orthodontic procedure.
